# Viralization as a microbial approach for enhancing coral reef restoration

**DOI:** 10.1093/ismejo/wraf110

**Published:** 2025-06-06

**Authors:** Jason Baer, Mark Little, Jenna Aquino, Anneke van der Geer, Andrés Sánchez-Quinto, Ashton Ballard, Catherine Lawrence, Jessica Carilli, Aaron Hartmann, Forest Rohwer

**Affiliations:** Department of Biology, San Diego State University, San Diego, CA 92182, United States; Department of Organismic & Evolutionary Biology, Harvard University, Cambridge, MA 01451, United States; Department of Biology, San Diego State University, San Diego, CA 92182, United States; Department of Biology, San Diego State University, San Diego, CA 92182, United States; Department of Biology, San Diego State University, San Diego, CA 92182, United States; Department of Biology, San Diego State University, San Diego, CA 92182, United States; Department of Biology, San Diego State University, San Diego, CA 92182, United States; Basic and Applied Research Division, Naval Information Warfare Center Pacific, San Diego, CA 92152, United States; Department of Organismic & Evolutionary Biology, Harvard University, Cambridge, MA 01451, United States; Perry Institute for Marine Science, Waitsfield, VT 05673, United States; Department of Biology, San Diego State University, San Diego, CA 92182, United States

**Keywords:** microbialization, coral restoration, microbial ecology, biogeochemistry, coral reef

## Abstract

Coral reef ecosystems rely on microorganisms to carry out biogeochemical processes essential to the survival of corals and the reef food web. However, widespread shifts from coral to algal dominance as a result of anthropogenic pressures have promoted microbial communities that compromise reef health through deoxygenation and disease. These degraded reefs become locked in a “microbialized” state characterized by high microbial biomass, low oxygen, and heightened pathogenic activity that stymie efforts to outplant corals onto the reef, a common approach applied to restore these ecosystems. Over 18 months, we compared viral and microbial dynamics alongside physical and chemical parameters (“water quality”) between two coral outplanting sites and two midwater reef mesocosms called Coral Arks. Seafloor sites exhibited microbialization, whereas Arks maintained conditions with higher viral abundances and virus-to-microbe ratios, smaller and less abundant microorganisms, and consistently higher dissolved oxygen, water flow, and light availability. These conditions, which we term “viralized”, supported enhanced coral growth and survival, greater benthic diversity, increased coral recruitment, reduced turf and macroalgae, and higher fish abundance compared to outplanting sites. Despite these benefits, analysis of microbial carbon metabolism genes revealed an underlying trend towards microbialization at both sites, reflecting larger-scale regional decline. These findings emphasize that microbial and physicochemical conditions are drivers of reef restoration outcomes; to be successful, restoration strategies must target the underlying environmental factors that support coral survival and resilience. We identify key microbial and physical variables—such as oxygen levels, flow, and viral activity—associated with viralized reef states, which should serve as focal points for developing new interventions and technologies aimed at creating conditions conducive to reef recovery.

## Introduction

Coral reefs are biodiversity hotspots, supporting an estimated 1.5 million species within or around the carbonate framework built by corals [1, 2]. However, reefs worldwide are experiencing severe declines due to climate change, overfishing, pollution, and habitat degradation, leading to widespread losses in coral cover and shifts in community composition [3, 4]. In response, restoration efforts have sought to slow or reverse these declines, but long-term success has been limited, particularly in degraded environments [5–7]. Many coral outplanting projects fail to establish self-sustaining populations, partly because they do not adequately address the environmental conditions driving coral decline. Without strategies to improve underlying reef conditions, or select locations for restoration conducive to coral survival, restoration efforts risk placing corals into environments where they will not survive as ecosystem functions continue to deteriorate.

Environmental conditions on coral reefs strongly influence microbial community composition, which in turn affects coral restoration outcomes. **Microorganisms drive nutrient cycling, organic matter processing, trophic interactions, and energy provisioning, all of which are key to reef stability and resilience** [8, 9]. Here we use ‘microorganisms’ to refer to free-living marine bacteria and archaea, which dominate microbial biomass and functional activity in reef seawater [9, 10]. On healthy reefs, free-living microorganisms play a central role in energy transfer through the microbial loop, where they consume dissolved organic carbon (DOC) and are themselves consumed, returning organic carbon and nutrients to the food web [9, 11]. This balance is maintained by viral lysis, protist grazing, and benthic suspension feeding, which regulate microbial populations and support high productivity in nutrient poor waters [9, 12, 13]. Healthy reefs are described as “viralized” systems due to the key role of viruses in controlling and connecting microorganisms to the trophic web [14, 15]. However, stressors such as eutrophication and overfishing disrupt microbial regulatory processes by promoting algal overgrowth and DOC enrichment, shifting microbial communities towards dominance by opportunistic and pathogenic taxa [16–18]. This process, known as microbialization, leads to uncontrolled microbial growth, depletion of dissolved oxygen (DO), and an increase in microbial pathogens [19–21], reshaping benthic conditions to favor non-calcifying organisms, such as algae, over corals [22]. Disturbances to benthic suspension feeders such as corals and sponges, which also consume free-living microorganisms and viruses, further exacerbate microbialization by disrupting the transfer of microbial carbon into higher trophic levels and weakening benthic-pelagic coupling [9, 23, 24].

Viral predation on microorganisms regulates reef microbial communities and influences whether a reef remains balanced or shifts towards microbialization [25, 26]. The virus-to-microbe ratio (VMR) is a useful indicator of viral control over microbial populations [14, 17, 27], with high VMRs reflecting strong viral predation through lysis, which limits microbial overgrowth and promotes community evenness [28–30]. In contrast, low VMRs signal a shift toward lysogeny, where viruses integrate into microbial genomes instead of lysing their hosts, allowing unchecked microbial growth [17, 31]. As reefs degrade, factors such as overfishing and eutrophication disrupt top-down and bottom-up controls on reef macroalgae, leading to increased DOC exudation that fuels the growth of copiotrophic and pathogenic microorganisms [32–35]. This shift further favors lysogeny, reducing viral predation and accelerating microbial expansion [17, 36]. The resulting microbial growth drives reef deoxygenation [20, 37], triggers disease outbreaks [18, 36, 38], and contributes to macroorganism morbidity and mortality [39, 40], reinforcing the transition to low-diversity, microbially dominated reef states. These feedback loops alter reef biogeochemistry and are major contributors to the high variability and low coral survival rates observed across restoration efforts [5, 7].

Evidence suggests that shifts in the coral-associated microbiome are a major cause of coral mortality during outplanting from nurseries to reef habitats [41, 42], yet the role of free-living seawater microorganisms in coral restoration remains less understood. Microbial community shifts driven by the release of DOC from the benthos [32, 36, 43] can rapidly and dramatically alter reef benthic communities through disease and deoxygenation [20, 40], even if the microbial shifts are short-lived [40, 44]. Microbial communities also respond dynamically to changes in reef conditions such as eutrophication, turbidity, and temperature [45], making them valuable predictors of restoration success. For instance, microbial communities on healthier, coral-dominated reefs tend to be dominated by autotrophic and oligotrophic taxa, such as SAR11 clade members and certain *Alphaproteobacteria* species, whereas highly impacted, algae-dominated reefs support a higher abundance of heterotrophs (*Gammaproteobacteria*, *Vibrionaceae*, *Pseudoalteromonadacaea*, *Bacteroidetes* species) and pathogens [19, 21, 43, 46]. Similar patterns occur in benthic foraminifera communities on healthy vs. degraded reefs [47, 48]. Algae-stimulated microbial communities exhibit lower diversity [17, 36], higher pathogenicity [18, 32], elevated metabolic rates [19, 49], and larger per-cell biomass [20, 50], shifting energy allocation toward microbial processes rather than corals and reef-associated macrofauna [51]. Addressing microbialization in restoration efforts by reducing macroalgal cover—through manual removal or herbivore enhancement—or by targeting areas with healthier microbial communities and improved water quality is likely to enhance coral survival and resilience.

**Figure 1:**
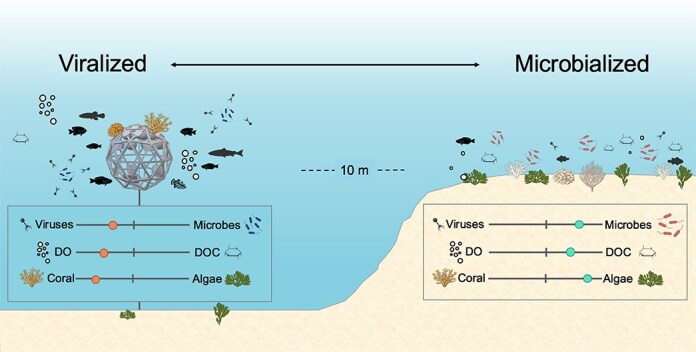
Hypothesis for the experiment. The viralized midwater environment was expected to support higher abundances of free viruses relative to microorganisms, higher DO and lower labile (bioavailable) DOC concentrations, and support coral survival over algae. The microbialized seafloor environment was expected to support a higher abundance of microorganisms relative to free viruses, higher labile DOC and lower DO concentrations, and reduced coral survival.

In this study, viral, microbial, physical, and chemical conditions were compared across two strategies for translocating corals: midwater Coral Reef Arks [52] and direct outplanting to reef substrate [53, 54]. Arks, installed offshore and elevated above the benthos, exhibited conditions more similar to open-ocean environments, whereas nearshore outplanting sites were influenced by terrestrial inputs and associated declines in water quality. Identical cohorts of coral fragments from eight species were translocated to both sites (see Carilli et al. 2024), and viral, microbial, physical, chemical, coral, algae, and fish metrics were monitored over ~18 months (Supplementary Figure S1). We hypothesized that the improved water quality and physicochemical conditions on midwater Arks would lead to fewer, smaller microorganisms and more free viruses, and that these viralized conditions would enhance coral survival (Fig. 1). Conversely, based on the higher algal abundance and more degraded condition of the reef areas, we expected that outplanting sites would exhibit microbialized conditions, with reduced viral lysis of microorganisms, higher microbial biomass, and poor coral survivorship.

## Materials and methods

### Sites

Two midwater Coral Reef Arks structures were installed off the west coast of Vieques, Puerto Rico in the northeastern Caribbean (Fig. S1). Identical cohorts of corals were outplanted to the Arks and to two seafloor outplanting sites used to emulate a commonly applied benthic restoration method (see Carilli et al. 2024 for more details).

### Arks structures

Coral Reef Arks (hereafter “Arks”) are positively buoyant midwater structures tethered to a seafloor anchoring system. Each Ark is a geodesic sphere measuring 2.4 m (8 ft) in diameter and constructed from stainless steel and fiberglass base materials following methods described in [52, 55]. In November 2021, two Arks were installed ~2 miles offshore of the west coast of Vieques Island, Puerto Rico (Fig. S1) within part of the U.S. Navy’s unexploded ordnance remediation site 16 (UXO 16). The installation site was characterized by sandy bottom at ~17 m depth (56 ft) with patches of seagrass and macroalgae (primarily *Padina* spp*.* and *Halimeda* spp*.*). Once installed, the midlines of the positively buoyant Arks were located at ~8.8 m depth (29 ft) and the tops of the Arks were located at ~7.6 m depth (25 ft).

### Outplanting sites

Two seafloor outplanting sites were selected off the west coast of Vieques at depths similar to the tops of the Arks structures to compare the Arks approach (midwater, offshore conditions) to a common method of outplanting corals to the seafloor (benthic, nearshore conditions) at the same depth. The two sites were located within another nearshore zone of UXO16 at 7.6 m (25 ft) and 6.4 m (21 ft) depth, respectively, and separated by ~25 m (Fig. S1). Outplanting sites were broadly characterized as reef hardbottom and included colonized pavement, linear reef, and aggregated patch reef habitats [56]. Descriptions of benthic composition at the outplanting sites were based on qualitative visual observations made during initial site surveys and coral outplanting activities. Similar to other reefs around Vieques, the outplanting sites were in relatively poor condition, with sparse living coral cover. Several genera of stony corals (*Orbicella, Siderastrea, Diploria,* and *Porites*) were present at the site, as well as abundant fire corals, soft corals, sponges, and a high benthic cover of turf and macroalgae. Patches of sand and seagrass were observed at the edges of the sites.

### Coral translocation

A total of 400 coral fragments representing eight species were distributed among the two Arks and two outplanting sites. Whereas the number of fragments per species was not equal (e.g. *Acropora* spp*.* had higher colony representation than *Orbicella* spp.), fragments were distributed so that each site received the same number of fragments from each species. In total, 100 coral fragments were moved to each site. Corals were sourced from a NOAA coral nursery on the northeast of Puerto Rico, from a sunken barge near Guayama on Puerto Rico’s south coast, and from a spalling concrete boat ramp at Mosquito Pier on Vieques’ north coast. Further details on coral species, attachment methods, and coral monitoring can be found in Carilli et al. (2024). The effects of the coral planting and associated macroorganism responses are not explored in this manuscript but are detailed in Carilli et al. (2024) and summarized in brief in Supplementary Fig. S2.

### Monitoring

Water samples were collected from the two Arks and two outplanting sites at three-month intervals over the course of a year for a total of six sampling events (November 2021, February 2022, May 2022, August 2022, December 2022, June 2023). Six water samples were collected from each site during each sampling event (n = 144 total) and analyzed for water chemistry (DOC, inorganic nutrients), viral and microbial abundances, microbial cell size, and microbial DNA. Sensors recorded additional time series data, including temperature, DO, flow speed, and light intensity, at each site for every monitoring event (detailed below). At each monitoring event, all translocated corals were measured and the percent of each coral that had suffered partial mortality (if any) and the percent of living tissue that was either healthy, pale, bleached, or disease was recorded. Photographs were collected to quantify algae cover, and fish communities were also surveyed at each event [57]. Details on coral health monitoring and fish community surveys are provided in Carilli et al. (2024).

### Water sample collection

Water samples were collected on SCUBA using 2 L polycarbonate Hatay-Niskin bottles as described in [52, 58]. Briefly, Hatay-Niskin bottles were rinsed prior to sampling with 5% hydrochloric acid solution (HCl) to remove organic carbon contamination, transported via SCUBA to the sampling site with the end caps of the bottles removed, passed back and forth to flush the interior of the cylinder with sample water, and sealed using end caps. At the outplanting sites, all water samples were collected from within 20 cm of the benthos, in close proximity to the translocated corals. Arks water samples were similarly collected in close proximity to the translocated corals, however half of the samples (n = 3) were collected from inside the Arks (below the corals) and half of the samples (n = 3) were collected from outside (above the corals) at each site. Water sampling occurred between the hours of 0900 and 1200 daily at all sites. Following sample collection, water samples were stored in the dark in a cooler until sample processing.

### Sample processing

Water samples were processed within 6 hours of collection to prepare for analysis of DOC; inorganic nutrients including nitrate + nitrite (NO_3_^−^ + NO_2_^−^), ammonia (NH_3_), and phosphate (PO_4_^3−^); enumeration of viral and microbial abundances; microbial cell size and biomass; and microbial DNA following protocols described previously (58).

### Viral and microbial abundances and VMR

Unfiltered sample water (1 ml) was fixed using paraformaldehyde (Electron Microscopy Sciences) to a final concentration of 2%, passed through a 0.02 μm Anodisc filter (Whatman), and stained with 5X SYBR Gold (Invitrogen). The filter was then mounted on a microscope slide using a mountant solution (glycerol, 1X PBS, ascorbic acid) and stored at −20°C until analysis via epifluorescence microscopy (excitation/emission: 325–375/537 nm wavelengths). Direct counts of microorganisms and viruses were obtained from microscope images using ImagePro Software (Media Cybernetics) and used to calculate viral and microbial abundances [59] and the VMR for each sample. Counts primarily reflect SYBR-stained free-living bacteria and archaea; phototrophic organisms such as cyanobacteria were not separately identified based on autofluorescence.

### Microbial biomass and cell size

Unfiltered sample water (1 ml) was fixed with glutaraldehyde (Electron Microscopy Sciences) to a final concentration of 0.3% v/v, passed through a 0.2 μm Anodisc filter (Whatman), and stained with 5 μg/ml DAPI (Molecular Probes, Invitrogen). The filter was then mounted on a microscope slide using a mountant solution (glycerol, 1X PBS, ascorbic acid) and stored at −20°C until analysis via epifluorescence microscopy (excitation/emission: 358/461 nm wavelengths). Microbial cell dimensions (length and width) were measured from microscope images using ImagePro Software (Media Cybernetics). Cell volume (μm^3^) was calculated from length and width measurements by assuming each cell has the shape of a cylinder with hemispherical endcaps, according to the following equation:


$$ V=\pi /4\times{w}^2\left(L\hbox{--} w/3\right) $$

Where *L* is length and *w* is width [50, 60]. Individual microbial cell volumes (μm^3^) were then converted to mass in wet weight (g) using previously established size-dependent relationships for marine microbial communities [50, 61] and summed to calculate total microbial biomass (g/10 m^3^) per water sample. Microbial biomass estimates primarily reflect free-living bacteria and archaea, as size measurements were limited to cells smaller than ~4 μm, effectively excluding larger microbial eukaryotes.

### DNA extraction and sequencing for microbial metagenomes

Microbial metagenomes were sequenced from water samples described above. One liter of each water sample was filtered through a 0.22 μm cylindrical Sterivex filter (EMD Millipore), dried, and stored at −20°C until laboratory processing. DNA was extracted from the filters using Nucleospin Tissue Extraction kits (Macherey Nagel), following the manufacturer's instructions with minor modifications: the amounts of T1 lysis buffer and proteinase K were doubled, and the lysis incubation time was increased to enhance DNA yield. Libraries were prepared using a xGEN ssDNA and low-input DNA Library Prep kit (IDT), with 100 ng of input DNA per sample standardized prior to library preparation. Shotgun metagenomic sequencing was then performed on a NovaSeq System (Illumina) with a 2 × 250 bp paired-end run configuration, sequenced at a depth of 10 million reads per sample. DNA extraction blanks (MilliQ water) were included as negative controls, processed through library preparation and sequencing alongside samples. Blanks yielded an average of ~5000 reads compared to ~10 million reads for environmental samples and were excluded from downstream analyses.

### Dissolved organic carbon

All glassware and tubing used were washed in 5% HCl to prevent organic carbon contamination. Sample seawater for bulk DOC analysis was filtered through a 0.22 μm Sterivex filter (EMD Millipore), discarding the first ~100 ml of filtered sample water. An acid-washed and pre-combusted 40 ml amber borosilicate glass vial was then rinsed five times with sample water and filled to the shoulder (~35 ml). The sample was then poisoned with three drops of full strength, molecular grade HCl, capped with a PTFE-lined silicone septa, and stored at 4°C until analysis via high-temperature catalytic oxidation [62].

### Inorganic nutrients

Sample seawater for inorganic nutrient analysis was filtered through a 0.22 μm Sterivex filter, discarding the first ~100 ml of filtered sample water. A clean, 20 ml HDPE plastic vial was then rinsed three times with sample water, filled to the shoulder (~18 ml), and frozen immediately at −20°C until analysis via flow injection analysis [63]. Certified calibration standards were used during flow injection analysis to generate standard curves for nitrate + nitrite, phosphate, and ammonia concentrations.

### Physical parameters

Current speeds (cm/s) were measured using an acoustic Doppler current profiler (Aquadopp 1 MHz, Nortek) mounted on a weighted milk crate and deployed upward-facing on the seafloor at the Arks and outplanting sites. Measurements were recorded at 5-minute intervals over ~2 days per site during each sampling event, with current speed data analyzed at 8 m depth for the Ark sites and 7.5 m depth for the outplanting sites. Dissolved oxygen (DO; mg/L and % saturation) was measured using pre-calibrated HOBO DO dataloggers (Onset), attached directly to the Ark structures with nylon cable ties or to stainless-steel stakes at the outplanting sites. DO measurements were recorded every 1 minute for ~4 days per site during each sampling event and were corrected for salinity using a portable refractometer. One DO sensor was deployed at each site.

Temperature (°C) and light intensity (lux) were measured using HOBO Pendant loggers (Onset) mounted upward-facing on small plates attached to the Arks or stakes at the outplanting sites. These sensors were deployed long-term at each site and recorded measurements every 5 minutes. However, algal fouling on the light sensors at both Arks and outplanting sites limited reliable light measurements to the first 2 weeks following deployment. To address this, sensors were manually cleaned at each sampling event, and only the first 10 days of light data following each cleaning were used in analyses.

### Data processing and statistical analysis

All data analyses were performed using *R* (Version 4.4.2) [64] and RStudio statistical software (Version 2024.12.0 + 467).

### Viral and microbial ecology

Statistical tests were performed on the variables VMR, viral-like particle (VLP) abundance, microbial abundance, mean microbial cell size, and total microbial biomass to evaluate differences between treatments (Ark and outplanting) and within treatments across time. Data normality was assessed using a Shapiro–Wilk test, and none of the variables were normally distributed. Accordingly, non-parametric tests were used throughout. Differences between the two treatments were assessed using Mann–Whitney U tests, both pooled across all time points and at each individual time point (0, 3, 6, 9, 12, and 18 months following deployment). To correct for multiple comparisons across time points, false discovery rate (FDR) adjustments were applied. Within each treatment, differences across time were evaluated using Kruskal-Wallis tests. An alpha level of 0.05 was used to assess statistical significance in all tests.

### Carbon metabolism gene pathways

All FASTQ files from microbial metagenomes were quality-filtered using Fastp and custom Python scripts [65]. Sequencing adapters were trimmed, and reads were globally trimmed by 20 bp from both ends. In addition, reads were subjected to quality trimming using a Phred score threshold of 30, meaning that any bases with a Phred score < 30 were removed from the ends of the reads until a base with a quality ≥30 was encountered. Reads were then filtered to remove duplicates, reads shorter than 200 bp after trimming, and reads with <30% complexity. After quality filtering, the shotgun metagenomic libraries generated an average of 9 140 263 reads per sample, with a mean read length of 250 bp ± 4.22 bp, indicating high sequencing depth across samples.

Sequence reads were mapped to the SEED database [66] for metabolic and taxonomic assignments using SUPERFOCUS, following methods described previously [67, 68]. Functional annotation was performed using only Read 1, as SUPERFOCUS is optimized for single-end reads and avoids merging-related artifacts. Taxonomic profiles were generated separately using the FOCUS pipeline, which classifies metagenomic reads based on k-mer profiles derived from complete bacterial and archaeal genomes in the NCBI RefSeq database [69]. The top 20 most abundant microbial taxa, based on relative abundance at the Arks and outplanting sites, are presented in Supplementary Table S7. All subsequent analyses focused on functional gene composition rather than microbial taxonomic profiles.

Temporal trends in microbial carbon metabolism strategies at Arks and outplanting sites were evaluated using sequencing data from five time points (0, 3, 6, 9, and 12 months). Six gene pathways were selected for analysis, each represented by genes encoding rate-limiting enzymes critical to central carbon metabolism as described previously [20]. The pathways included Anaplerosis (reactions replenishing TCA cycle intermediates), the Embden-Meyerhof-Parnas (EMP) pathway, the Entner-Doudoroff (ED) pathway, the Pentose Phosphate Pathway (PPP), the Oxidative Krebs cycle, and reactions fueling ED and PPP. Normalized gene hits (hits per 100 000 reads) were used as proxies for the relative activity of each pathway within microbial communities.

Linear mixed-effects models were applied to evaluate how microbial carbon metabolism pathways changed over time and differed between treatments (Ark vs outplanting). For each of the six pathways, models included fixed effects for time, treatment, and their interaction, with sample identity included as a random effect to account for repeated measures. Type III ANOVA was used to assess the significance of the interaction terms, testing whether temporal trends differed between treatments. A separate model was used to test the overall effect of treatment across all time points. To further explore pathway dynamics, temporal trends within each treatment group (Ark and outplanting) were analyzed separately using mixed-effects models with time as a fixed effect and sample identity as a random effect, and visualized with separate regression lines for Ark and outplanting treatments. All *P* values were corrected for multiple comparisons using the FDR method.

### Physical and chemical parameters

Dissolved oxygen, temperature, flow, and light intensity data were collected as time series. For the DO and light intensity variables, measurements were classified as “night” or “day” measurements based on the sensor timestamp (between 7 p.m. and 7 a.m. local time for night, and between 7 a.m. and 7 p.m. local time for day). Only daytime measurements of light intensity were used in statistical analyses. Maximum Daytime Dissolved Oxygen (MaxDDO) for each time point was calculated by averaging the top 5% of daytime DO (% saturation) values at each site. Similarly, Minimum Nighttime Dissolved Oxygen (MinNDO) for each time point was calculated by averaging the bottom 5% of nighttime DO (% saturation) values at each site, thus capturing the maximum range of DO experienced at each site and timepoint. The DO saturation ratio (DO sat ratio) was calculated by dividing MaxDDO by MinNDO to generate a single value describing the diel variance in DO for each site and time point.

All time series data were tested for normality using a Shapiro-Wilks test and for homogeneity of variance using a Levene’s test. In all cases, data were not normally distributed and had unequal variances between groups. Consequently, non-parametric tests were used for all analyses. To compare treatments (Ark vs outplanting) at individual time points, Mann–Whitney U tests were used. To assess overall treatment differences in DO, temperature, flow, and light intensity across all time points, Kruskal-Wallis tests were employed, as they allow comparison of more than two groups (i.e. treatment × time combinations) without assuming normality or equal variance. FDR correction was applied to account for multiple comparisons.

Similar statistical tests were performed on DOC, ammonia, nitrate + nitrite, and phosphate as for microbial ecology variables. For inorganic nutrients (nitrate + nitrite, phosphate, and ammonia), raw concentration values were retained as reported by the analytical instrument; no values were set to zero. Values falling below the laboratory method detection limit were low but nonzero and were included directly in all statistical analyses as recommended previously [70]. No chemistry data were collected at the initial time point due to sampling constraints, but for each successive monitoring time point (3, 6, 9, 12, and 18 months following deployment), comparisons between treatments were made using non-parametric Mann–Whitney U tests. Between-treatment differences at individual time points were also assessed using Mann–Whitney U tests. Within each treatment, changes in chemistry variables over time were evaluated using Kruskal-Wallis tests. All *P* values were FDR-adjusted to account for multiple comparisons.

### Variable relationships and multivariate analyses

All variables described above were averaged to a single value per site per time point to simplify between-metric comparisons. A Spearman’s correlation matrix was used to test for significant correlations between the variables. Each correlation coefficient was interpreted to understand the strength and direction of the monotonic relationship between pairs of variables, where values close to ±1 indicate strong relationships, and values close to 0 indicate weak or no relationship. A heatmap of the correlation matrix was generated with a color gradient indicating the strength and sign of each correlation (Fig. S3). Linear regression models were performed on several of the strongly correlated variables, including (i) viral abundance and microbial abundance, (ii) VMR and MinNDO, (iii) viral abundance and DOC, and (iv) VMR and total microbial biomass. T-tests were performed on the coefficients of each linear regression model to test for significance.

The same dataset was used in a supervised random forest analysis to identify the most significant predictors of whether samples were collected from Arks or outplanting sites. The first sampling time point was retained to maximize statistical power, and missing chemistry data from this time point were imputed using the missForest package in *R*. A permuted random forest was then used to identify statistically significant predictors (Fig. S5). PCA was then performed on the imputed dataset to identify patterns of variability and to reduce the dimensionality of the data, including all normalized variables from the collected datasets. The number of principal components retained was determined based on the Kaiser criterion, retaining only components with eigenvalues greater than 1, and this was further supported by examination of a scree plot showing a clear elbow. The resulting components were interpreted based on their loadings, with high absolute values indicating variables that contributed most to each component.

## Results

### Viral and microbial ecology

**Figure 2:**
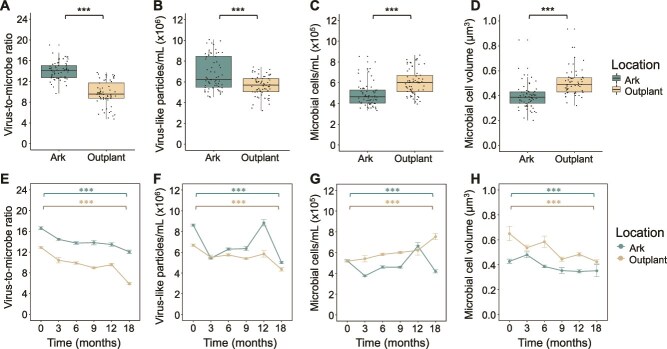
Viral and microbial ecology variables at Arks and outplanting sites. (A-D) Boxplots showing VMR, VLP abundance, microbial abundance, and mean microbial cell volume of seawater microbial communities collected from the Arks and outplanting sites. Asterisks denote significant differences between Ark and outplanting treatments (Mann–Whitney U test, FDR-adjusted *P* < .001). (E-H) Line plots showing the same variables as in (A-D) plotted over time (months following deployment). Colored asterisks denote significant differences within the Ark (teal) and outplanting (tan) treatments over time (Kruskal-Wallis test, FDR-adjusted *P* < .001).

Over the 18-month experiment, characteristics of viral and microbial communities at the Arks differed significantly from those at the outplanting sites at nearly all time points (Supplementary Table S1). The Arks supported communities with significantly higher virus-to-microbe ratios (Fig. 2A; Ark mean = 14.06, outplanting mean = 9.71; n = 130) and VLP abundances (Fig. 2B; 6.82 vs. 5.62 × 10^6^/ml; n = 130), and significantly lower microbial cell abundances (Fig. 2C; 4.86 vs. 6.03 × 10^5^/ml; n = 131), mean microbial cell volumes (Fig. 2D; 0.39 vs. 0.50 μm^3^; n = 121), and total microbial biomass (Supplementary Fig. S2; 2.25 vs. 3.30 g/10 m^3^; n = 121) compared to the outplanting sites (Mann Whitney U tests, all FDR-adjusted *P* < .001; Supplementary Table S1). Viral and microbial metrics also varied significantly over time within both the Arks and outplanting sites, potentially reflecting seasonal changes or natural variability. These differences were significant for VMR (Fig. 2E; n = 65), VLP abundance (Fig. 2F; n = 65), microbial cell abundance (Fig. 2G; n = 65), mean microbial cell volume (Fig. 2H; n = 56), and total microbial biomass (Supplemental Fig. S2; n = 56) for both the Arks and outplanting treatments (Kruskal-Wallis test, all FDR-adjusted *P* < .001; Supplementary Table S3).

### Physical and chemical variables

**Figure 3:**
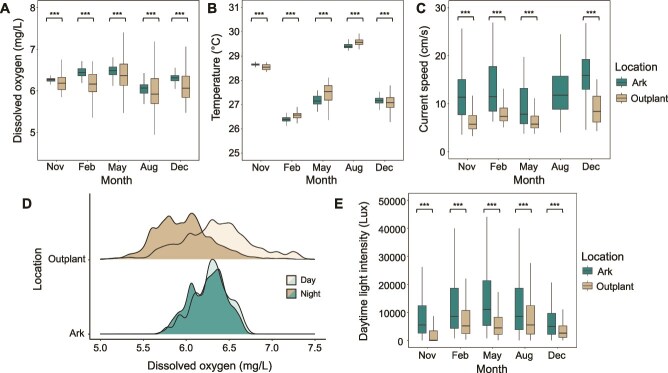
Physical variables at Arks and outplanting sites. (A-C, E) Boxplots showing DO, temperature, average current speed, and daytime light intensity measured at the Arks and outplanting sites during each monitoring time point. Asterisks denote significant differences between Ark and outplanting treatments at each time point (Mann–Whitney U test, *P* < .001). (D) Ridgeline plot showing the diel range of DO measurements collected at Arks and outplanting sites, with daytime measurements displayed with light shading and nighttime measurements displayed with darker shading.

The Arks and outplanting sites consistently displayed significant differences in physical parameters. Over all time, DO concentrations (Fig. 3A; Ark mean = 6.26 mg/L, outplanting mean = 6.15 mg/L), current speeds (Fig. 3C; 12.98 vs. 7.47 cm/s), and daytime light intensities (Fig. 3E; 10 252 vs. 5493 lux) were significantly higher at the Arks than at the outplanting sites (Kruskal-Wallis test, all FDR-adjusted *P* < .001; Supplementary Table S5). In contrast, mean water temperatures did not differ significantly between treatments when compared across all time points (Fig. 3B; Ark mean = 28.29°C, outplanting mean = 28.31°C; Kruskal-Wallis test, FDR-adjusted *P* = .762). Despite the lack of a significant overall temperature difference, all four physical variables—including temperature—differed significantly between the two treatments at every individual monitoring time point (Mann–Whitney U tests, all FDR-adjusted *P* < .001; Supplementary Table S4).

**Figure 4:**
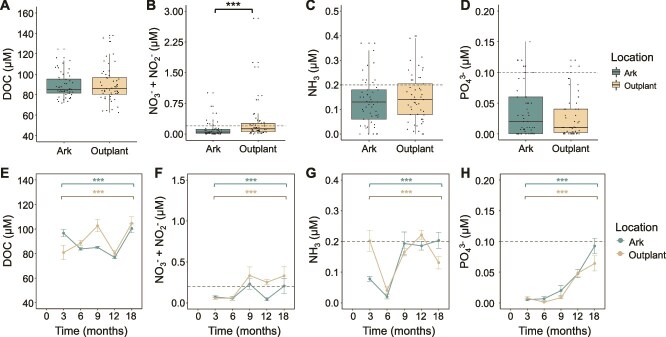
Chemical variables at Arks and outplanting sites. (A-D) Boxplots showing DOC, nitrate + nitrite (NO_3_^−^ + NO_2_^−^), ammonia (NH_3_), and phosphate (PO_4_^3−^) concentrations in seawater collected from the Arks and outplanting sites. Asterisks denote significant differences between Ark and outplanting treatments (Mann Whitney U test, FDR-adjusted *P* < .01). Gray dashed line indicates the laboratory method detection limit (MDL) for nutrient analysis. (E-H) Line plots showing the same variables as in (A-D) plotted over time (months since deployment). Colored asterisks denote significant differences within the Ark (teal) and outplanting (tan) treatments over time (Kruskal-Wallis test, FDR-adjusted *P* < .01).

Nutrient and DOC concentrations tracked similarly across both the Ark and outplanting sites, with significant differences between treatments limited to a few individual time points (Supplementary Table S1). Over all time points, DOC concentrations (Fig. 4A; Ark mean = 89.01 μM, outplanting mean = 91.24 μM; n = 107), phosphate (PO_4_^3−^; Fig. 4D; 0.03 vs. 0.02 μM; n = 106), and ammonia (NH_3_; Fig. 4C; 0.13 vs. 0.15 μM; n = 106) did not differ significantly between treatments (Mann–Whitney U tests, all FDR-adjusted *P* > .05; Supplementary Table S1). However, nitrate + nitrite (NO_3_^−^ + NO_2_^−^) concentrations were significantly higher at the outplanting sites (Fig. 4F; Ark mean = 0.11 μM, outplanting mean = 0.5 μM; n = 106; FDR-adjusted *P* < .001). When comparing treatments at individual time points, DOC was significantly lower at the outplanting site at 3 months and significantly higher at 9 months (Fig. 4E), ammonia was higher at the outplanting site at 3 months (Fig. 4G), and nitrate + nitrite was higher at the outplanting sites at 12 and 18 months (Fig. 4F) (Mann Whitney U tests, all FDR-adjusted *P* < 0.01). Each of these variables also varied significantly over time within both the Ark and outplanting treatments, indicating temporal shifts in water chemistry at each site (Fig. 4E-H; Kruskal-Wallis test, all FDR-adjusted *P* < .001; Supplementary Table S3).

### Variable relationships and multivariate analyses

**Figure 5:**
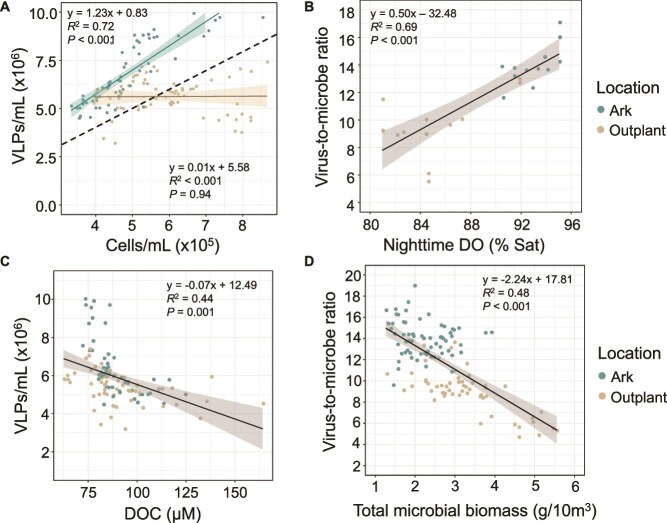
Linear regression models of select viral, microbial, chemical, and physical variables at Arks and outplanting sites with linear equations, *R*^2^, and *P* value (t-test) included. (A) VLP abundance vs. microbial cell abundance, with separate linear regression models for Arks and outplanting sites. The black dashed line indicates a 10:1 ratio, shown to reflect the expected VMR in the absence of density-dependent growth relationships [16]. (B) Virus-to-microbe ratio vs average nighttime DO. (C) VLP abundance vs DOC concentration. (D) Virus-to-microbe ratio vs total microbial biomass.

Spearman’s rank correlation coefficients were computed and plotted on a correlation matrix to assess the monotonic relationships between viral and microbial ecology and physicochemical variables (Supplementary Fig. S4). Several significantly correlated variable pairs were evaluated using linear regression analysis. At the Arks, VLP abundance and microbial abundance were significantly positively correlated (Fig. 5A; *R*^2^ = 0.72, *P* < .001), but the same relationship was absent at the outplanting sites (Fig. 5A; *R*^2^ = < 0.01, *P* = .94). Across all sites, VMR was significantly positively correlated with nighttime DO (MinNDO; Fig. 5B; *R*^2^ = 0.69, *P* < .001) and significantly negatively correlated with total microbial biomass (Fig. 5D; *R*^2^ = 0.48, *P* < .001). VLP abundance was significantly negatively correlated with DOC concentration (Fig. 5C; *R*^2^ = 0.44; *P* < .01).

**Figure 6:**
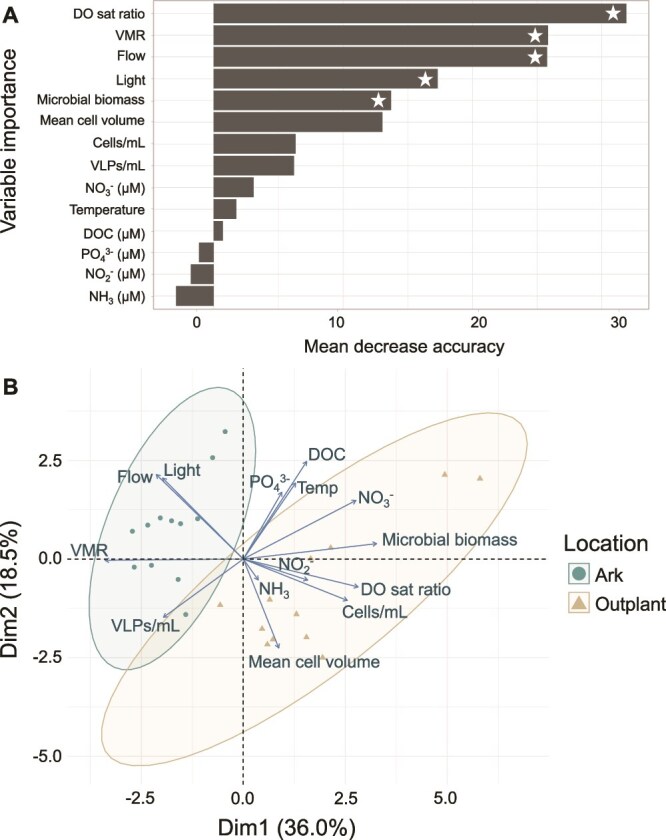
Multivariate analyses of viral and microbial, chemical, and physical variables at Arks and outplanting sites. (A) Supervised random forest analysis showing variables most predictive of the Ark vs outplanting treatments. White stars indicate significant variables as identified using permuted random forest. (B) Principal coordinate analysis (PCA) biplot showing variables driving the variance between Ark and outplanting treatments.

To evaluate which of these measured physicochemical variables were the most significant drivers, a supervised random forest analysis using 4000 trees was performed to identify the variables that best predicted the Ark versus outplanting site environments. The model had an out-of-bag error rate of 11%, meaning ~89% of samples were correctly classified during internal cross-validation (Fig. 6A). This permuted random forest analysis identified DO sat ratio (the ratio of daytime to nighttime DO; see Methods) as the most significant predictor, followed by VMR, flow speed, daytime light intensity, and total microbial biomass (Fig. 6A, permuted random forest, all *P* < .05).

This variability was also visualized using principle component analysis (PCA), which explained 54.5% of the variance within the dataset with two components: Dim1 (36.0%) and Dim2 (18.5%). The PCA biplot (Fig. 6B) showed distinct clustering of samples from Arks and outplanting sites, with Arks samples significantly separated from outplanting samples (PCA, *P* < 0.05). High loadings for key variables along Dim1 again indicated strong contributions of the same variables: microbial biomass, DO sat ratio, VMR, flow, and light, with more minor contributions from other variables.

**Figure 7:**
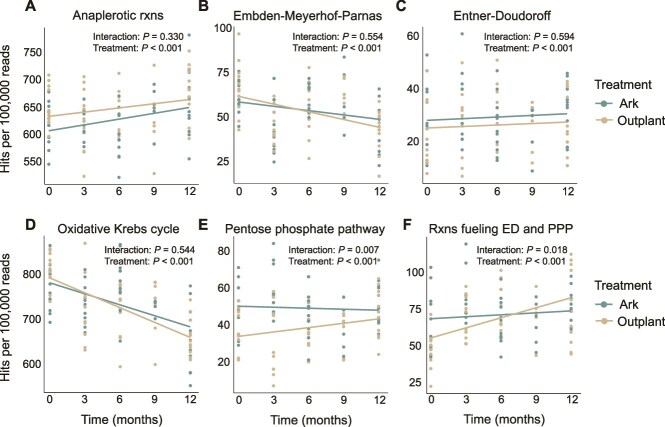
Temporal trends in central carbon metabolism gene pathways within microbial communities at Ark (teal) and outplanting (tan) sites. Each datapoint represents the number of hits per 100 000 sequencing reads for a single water sample metagenome. Six pathways are shown: (A) Anaplerotic reactions [rxns] (replenishing the oxidative Krebs [TCA] cycle intermediates), (B) the energy-efficient EMP glycolytic pathway, (C) the less energy-efficient ED glycolytic pathway, (D) TCA cycle, (E) the PPP, and (F) reactions [rxns] fueling ED and PPP. Solid lines represent trends based on linear mixed-effects models. FDR-adjusted *P* values from Type III ANOVA are shown for both the main effect of treatment and the treatment × time interaction effect.

Metagenomic analysis revealed differences in central carbon metabolism gene pathways between the Arks and outplanting sites, reflecting differences in how energy is cycled by microorganisms at each site. Linear mixed-effects models and Type III ANOVA showed that all six pathways—anaplerotic reactions, the EMP pathway, the ED pathway, the PPP, the oxidative Krebs cycle, and reactions fueling ED and PPP—changed significantly over time within both the Ark and outplanting treatments (Type III ANOVA, all FDR-adjusted *P* < .001; Supplementary Table S6). When averaged across all time points, these pathways also differed significantly between the two treatments (Fig. 7; all FDR-adjusted *P* < .001), indicating divergence in metabolic gene representation between Ark and outplanting site microbial communities (Supplementary Table S6).

Only two pathways exhibited significant interaction effects between time and treatment, meaning the slope of temporal change differed between Arks and outplanting sites. Gene pathways involved in the PPP (Fig. 7E; interaction *P* = .007) and reactions fueling ED and PPP (Fig. 7F; interaction *P* = .018) showed significantly different trajectories over time between treatments. No significant interaction effects were observed for the more energy-conserving EMP pathway (Fig. 7B; interaction *P* = .544), the less energy-conserving ED pathway (Fig. 7C; interaction *P* = .594), anaplerotic reactions (Fig. 7A; interaction *P* = .330), or the oxidative Krebs cycle (Fig. 7D; interaction *P* = .544; Supplementary Table S6). Hits for the EMP pathway and oxidative Krebs cycle decreased in both treatments over time, with a sharper decline in outplanting samples, suggesting that Ark communities retained higher representation of microorganisms utilizing these pathways. In contrast, hits for anaplerotic reactions and reactions fueling ED and PPP increased over time in both treatments.

## Discussion

This study characterized the viral, microbial, chemical, and physical environments associated with two distinct strategies for coral restoration in reef ecosystems. We compared midwater Coral Arks to seafloor outplanting sites located closer to shore but at similar depths. Viral and microbial communities at the Arks resembled healthy, viralized reef environments, with higher VMRs, more abundant free viruses (a key microbial control mechanism), and smaller, less abundant microorganisms compared to the more microbialized outplanting sites (Fig. 2). The Arks displayed higher DO concentrations (especially at night), flow speeds, and light intensities relative to the seafloor sites (Fig. 3), all of which impact fundamental growth and metabolic processes in corals and other reef macroorganisms [71, 72]. These favorable conditions persisted throughout the 18-month study—even as biological growth and ecological succession progressed on the Arks—and were associated with significantly improved coral survival and ecological function compared to the seafloor sites [57].

### Coral survivorship, recruitment, and benthic community structure

We predicted that microbialized conditions at the seafloor outplanting sites would result in poor survivorship among translocated corals. Carilli et al. (2024) tracked coral growth, survival, fish communities, and algae cover at the Arks and outplanting sites over the same timeline as this study. Coral fragments from eight species (n = 200 per treatment) survived and grew significantly better at the Arks, with 47% of corals surviving after 18 months compared to 24% at the outplanting sites [57]. Coral recruitment was observed on the experimental tiles at the Arks, whereas no recruits were found on equivalent tiles at the outplanting sites, indicating a more favorable environment on the Arks for both translocated and naturally settling corals. Limestone plates with coral fragments at the seafloor sites became rapidly fouled by turf and fleshy algae, a key driver of microbialization associated with pathogenic microbial communities that promote coral disease and reduce restoration success [43, 73, 74]. In contrast, plates at the Arks had less algae cover and were colonized by diverse invertebrate communities including sponges, bryozoans, hydrozoans, and crustose coralline algae [57]—the latter being a known inducer of coral settlement [75]. The fish communities at the Arks became dominated by higher trophic level fish communities, with higher biomass and abundance of piscivorous fish after nine months [57]. This top-heavy trophic structure is characteristic of healthier reefs and contrasts with degraded reefs [76, 77], where declines in predators and shifts toward herbivore-dominated fish communities are common.

### Physicochemical conditions—water quality and biogeochemistry

Physical and chemical parameters played key roles in shaping microbial and viral community structure, as well as coral growth, survival, and benthic succession at the Arks and outplanting sites. The Arks experienced higher flow speeds (Fig. 3C), which are known to increase coral growth rates [78–80], enhance resilience to bleaching [71], and suppress turf and macroalgae cover [81]. Improved water clarity offshore provided corals with more light for photosynthesis and growth compared to the more turbid nearshore environment (Fig. 3E), where turf algaebetter adapted to low-light conditions—had a competitive advantage [82]. Although average DO concentrations were only slightly higher at the Arks, the diel range of DO was substantially larger at the outplanting sites (Fig. 3A). Supersaturated DO levels during the day reflect algal photosynthesis, whereas nighttime depletion indicates respiratory drawdown, a pattern frequently observed on degraded reefs [83–85]. Such nighttime oxygen lows negatively impact the growth and survival of corals, mobile invertebrates, and reef fish [72], and has been linked to microbial shifts favoring opportunistic and pathogenic species [40]. Temperature differences between the Arks and outplanting sites when pooled across time were not statistically significant, but differed significantly at each time point (Fig. 3B). However, the magnitude of the differences were small and unlikely to have meaningfully contributed to differential thermal stress at either site.

Dissolved organic carbon (DOC) and seawater nutrient levels did not significantly differ between the Arks and outplanting sites, nor were they strong predictors of reef condition in PCA or random forest analysis (Fig. 6). This was somewhat unexpected, given that these variables are often cited as strong predictors of microbialization [21, 34, 35]. However, this may reflect the rapid uptake of nutrients and DOC into algae and microbial biomass, which results in low dissolved concentrations of these chemicals even at reef sites with high rates of input [85–87]. Among inorganic nutrients, ammonia and nitrate + nitrite concentrations were slightly higher at the outplanting sites, but overall nutrient concentrations remained low at both locations, with 90%, 69%, and 71% of phosphate, nitrate + nitrite, and ammonia measurements falling below the laboratory method detection limit, respectively (Fig. 4F-G). These detection limitations constrain our ability to fully characterize nutrient dynamics, and observed concentrations likely represent minimum estimates of true environmental availability. The slight increase in ammonia at the Arks after 9 months may be linked to greater fish biomass [57] and associated nitrogen-rich waste products [88].

DOC concentrations were significantly different between treatments at only two time points (Fig. 4E). DOC has been experimentally demonstrated to drive microbialization on reefs [19, 33, 34], but its role remains difficult to disentangle due to limitations in our ability to selectively measure the labile, or bioavailable, fraction [89]. Labile DOC is readily consumed by microorganisms within minutes to hours, while refractory DOC persists longer in the water column and is more resistant to degradation [90]. These results underscore a broader challenge in microbial ecology to resolve DOC composition in order to fully understand its influence on microbial community structure and metabolic activity. The lack of differentiation between Arks and outplanting sites may reflect rapid microbial uptake of labile DOC, leaving behind a refractory DOC signal that does not capture dynamic microbial processes. Further research is needed to develop methods for resolving labile DOC dynamics *in situ* and linking them to microbial responses in reef environments.

### Viralization and microbialization dynamics

Viralization at the midwater Arks was associated with higher coral survivorship and overall ecosystem health, as microbial communities were better integrated into the trophic web. Microbialized reefs typically have lower VMRs than healthy reefs, indicating reduced viral lytic control and resulting microbial overgrowth [14, 17]. In contrast, the higher VMR and free virus abundance at the Arks (Fig. 2A, 5A) indicate a more balanced microbial community, aligning with the Kill-the-Winner model of viral ecology [30]. Under this model, viral predation limits the microbial standing stock, suppresses the rise of dominant copiotrophs, turns over inorganic nutrients, and promotes microbial community evenness [13, 91]. At the outplanting sites, reduced free virus abundance relative to microorganisms (Fig. 5A) suggests a shift toward lysogeny, consistent with the Piggyback-the-Winner model—where at high microbial densities, viruses opt to integrate into microbial genomes instead of lysing their hosts [17, 92]. Supporting this, negative correlations between viral abundance and DOC (Fig. 5C) and between VMR and total microbial biomass (Fig. 5D) suggest that microbial expansion at outplanting sites was fueled by organic matter availability and reinforced by shifts in viral infection strategies. Labile DOC is rapidly consumed by microorganisms [10, 85, 93], leading to higher microbial densities that drive free viruses to favor lysogeny over lysis, thus weakening viral predation and intensifying microbialization. A strong positive correlation between VMR and DO (Fig. 5B), both key predictors of reef state (Fig. 6A), suggests that oxygen enhances viral lytic activity, potentially by increasing microbial intracellular ATP concentrations and promoting viral replication [20, 31, 94]. These findings indicate that stabilizing microbial communities through enhanced viral predation is a key mechanism for improving coral survival and reef resilience. Additionally, microbial community structure and virus–microorganism dynamics may have been influenced by grazing from microbial eukaryotes (e.g. flagellates, ciliates) and potential trophic cascades involving higher trophic levels, as observed in other marine systems [95].

### Microbial metabolism and regional trends

Shifts in microbial carbon metabolism pathways provide further insight into microbialization trends at the Arks and outplanting sites. Previous studies have shown that exposure to labile DOC drives microbial communities to shift from slower, energy-conservative (high ATP-yielding) glycolytic pathways such as the EMP pathway to faster but less conservative (lower ATP yield) pathways like the ED and PPPs [20]. This shift enables microorganisms to rapidly exploit abundant and bioavailable carbon sources at sites with more DOC but comes at the cost of incomplete oxidation, diverting excess carbon into biomass and increasing microbial cell size [20, 49, 50]. In this study, genes encoding the EMP pathway and oxidative Krebs cycle declined over time at both sites, while genes associated with the ED pathway, PPP, and anaplerotic reactions increased over time (Fig. 7). The decrease in energy-conserving pathways and increased reliance on alternative metabolic routes at both sites indicate a broader regional trend towards microbialization over the study period, also reflected in decreasing VMRs and other metrics temporally (Fig. 2). These shifts are consistent with observed reef decline across the Caribbean, as microbial communities increasingly adapt to degraded, organic matter-rich conditions by prioritizing rapid, but inefficient carbon metabolism. Although both sites showed signs of microbialization, microorganisms at the Arks retained a higher representation of energy-conserving metabolic pathways and smaller average cell size (Fig. 2D), suggesting that viral regulation and improved water quality at the Arks buffered microbial shifts toward degradation.

### Restoration implications

Significant differences in the microbial and physical environment between the Arks and outplanting sites highlight the potential for targeted interventions to improve coral restoration outcomes. Effective restoration strategies must address the environmental drivers of microbialization to create conditions that support coral recovery. Reducing nutrient pollution from agricultural runoff, sewage discharge, and coastal development [96, 97], while protecting herbivorous fish through fisheries management and marine protected areas can help control macroalgal cover and reduce DOC exudates that stimulate microbialization [98, 99]. Active measures to control macroalgae, such as enhancing populations of key grazers (e.g. *Diadema* sea urchins in the Caribbean) or manually removing macroalgae, could further buffer the microbial feedback loops that macroalgae initiate [98, 100, 101]. Coral outplanting efforts should be paired with improvements in water quality to increase success. Deploying midwater Coral Arks, which provide more stable microbial and physicochemical conditions that support coral settlement and persistence, could be one way to achieve improved water quality conditions for outplanted corals. Additionally, monitoring microbial communities can serve as an early warning system for environmental stress and disease outbreaks [45], guiding interventions before large-scale mortality occurs. Emerging microbial-based approaches, such as phage therapy and probiotics [102, 103], offer additional tools for stabilizing reef microbiomes and mitigating microbialization-driven coral loss and are important areas of future research. Over the longer term, these results point to key environmental variables, such as DO, water flow, and viral activity, that should be considered in the design of large-scale interventions aimed at reshaping degraded sites towards conditions more favorable for coral reef recovery [104].

## Conclusions

This study demonstrates that translocated corals survive better in viralized environments with improved water quality, with VMR and DO emerging as the most influential factors defining healthy sites for corals. By maintaining higher VMR and DO levels, the midwater Arks provided conditions that enhanced coral persistence, in contrast to lower survival rates on the seafloor, underscoring the key role of microbial and physicochemical conditions in coral restoration success. Despite its significant impact, microbialization remains an overlooked challenge in coral reef restoration, which may explain the limited success of outplanting projects in degraded environments. Our findings show that simply translocating corals into compromised conditions is insufficient—restoration efforts must actively improve microbial and environmental parameters to achieve success. Whereas the Arks’ more favorable viral, microbial, and physicochemical conditions supported rapid ecological succession with diverse assemblages of biota and supported coral recruitment, the microbialized outplanting sites were quickly overgrown by turf and macroalgae and lacked successful coral recruitment. To enhance restoration success, strategies should incorporate microbialization dynamics into project design. This could include relocating restoration efforts to areas with lower microbialization risk, deploying elevated structures like Coral Arks, floating nurseries [105], or Mars Stars [106], and using microorganisms as diagnostic tools for site selection [45, 107]. Additionally, addressing nutrient pollution, protecting herbivorous fish populations, removing macroalgae through biocontrol or manual interventions, and monitoring microbial communities could help mitigate microbialization at a broader scale. By integrating these strategies, restoration efforts can move beyond translocating corals into failing environments and instead create conditions that actively support reef resilience and recovery.

## Supplementary Material

ArksViralization_Supplemental_FINAL_wraf110

## Data Availability

The raw sequence reads generated in this study from microbial shotgun metagenomes have been deposited in the NCBI Sequence Read Archive under BioProject accession number **PRJNA1257468**. All data are publicly available and can be accessed at: https://www.ncbi.nlm.nih.gov/bioproject/PRJNA1257468. Associated metadata, including geographic coordinates, sample collection dates, and environmental context, as well as processed gene count matrices and functional annotation tables, are available via Zenodo at doi:10.5281/zenodo.15313922. All relevant analysis scripts and bioinformatics workflows are available upon request and will be made publicly accessible upon publication.
