# Supplementary material for: Viralization as a microbial approach for enhancing coral reef restoration
Source: ISME J. 2025 Jun 6;19(1):wraf110. doi: 10.1093/ismejo/wraf110 (PMC12198774; doi:10.1093/ismejo/wraf110)
Supplement: ArksViralization_Supplemental_FINAL_wraf110 [file arksviralization_supplemental_final_wraf110.pdf]

Supplemental material for "**Viralization as a microbial approach for enhancing coral reef restoration**"

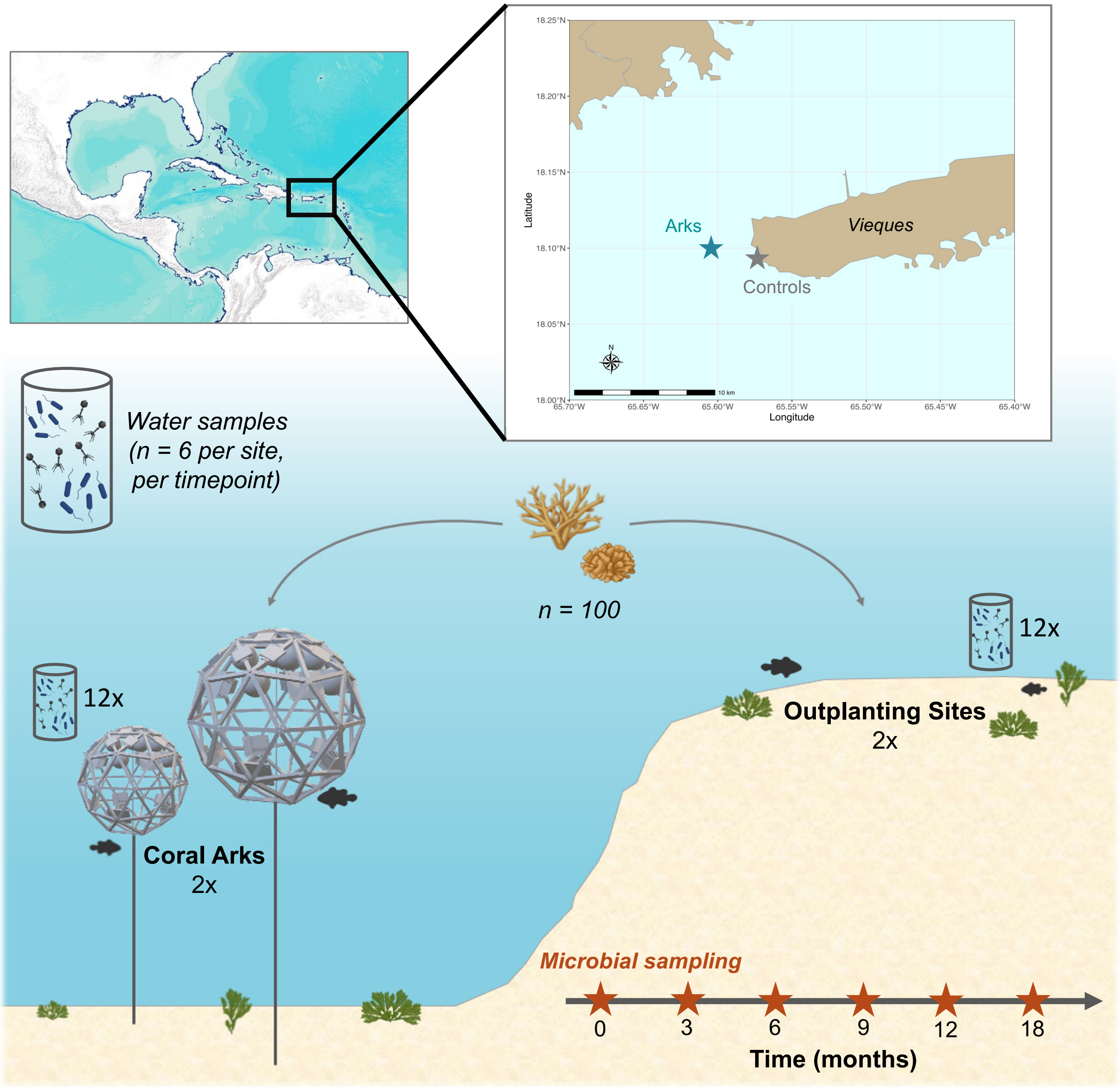

Figure S1. Experimental design and water sampling scheme. The experiment was conducted offshore of Isla Vieques, Puerto Rico, located within the eastern Caribbean Sea *(top left map)*. Two Coral Arks were deployed approximately 2 miles offshore of Isla Vieques and two outplanting sites were installed at the same depth as the Arks, but closer to shore *(top right map)*. Coral fragments (n = 100 per site) were distributed equally across both Arks and outplanting sites at the beginning of the experiment (0 months on the timeline). 6 water samples were collected from each Ark and outplanting site during each of 6 monitoring events (total 144 samples).

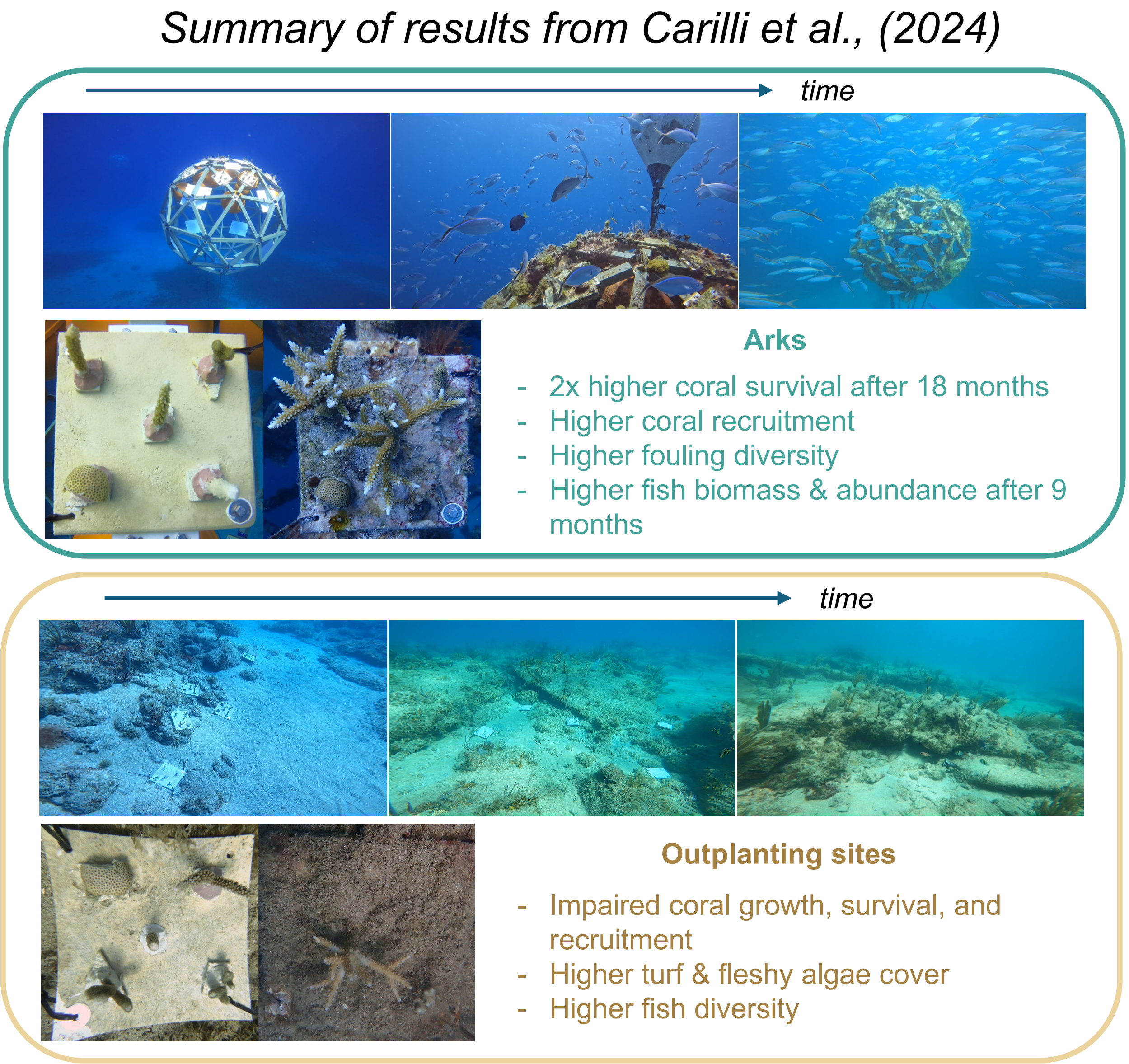

Figure S2. Summary of key findings from Carilli et al. (2024), which followed the same experimental timeline and design as the current study. That work focused on macroorganism responses to coral translocation and reported higher coral survival, recruitment, fouling diversity, and fish biomass at the Arks, as well as greater turf and fleshy algal cover at the outplanting sites. These complementary results provide ecological context for the microbial and physicochemical dynamics presented in this manuscript.

Table S1. Results of Mann–Whitney U tests comparing viral, microbial, and chemistry variables between Arks and outplanting treatments, pooled across all time points. For each variable, a non-parametric Mann–Whitney U test was used to assess differences in distributions between treatments. False discovery rate (FDR)–adjusted *P* values are reported, with significant values (α = 0.05) shown in bold. The number of observations (n) and group means for Arks and outplanting sites are also included.

Variable	**Test type**	**Grouping**	**n**	**Mean Ark**	**Mean Outplant**	***P* value (FDR-adjusted)**
Virus-to-microbe ratio	MW	All time	130	14.06	9.71	**< 0.001**
Virus-like-particles/mL	MW	All time	130	6.82	5.62	**< 0.001**

Microbial cells/mL	MW	All time	131	4.86	6.03	**< 0.001**
Microbial cell volume	MW	All time	121	0.39	0.50	**< 0.001**
Total microbial biomass	MW	All time	121	2.25	3.30	**< 0.001**
DOC	MW	All time	107	89.01	91.24	0.911
PO_4^{3-}	MW	All time	106	0.03	0.02	0.627
$NO_3^- + NO_2^-$	MW	All time	106	0.11	0.50	**< 0.001**
NH_3	MW	All time	106	0.13	0.15	0.337

Table S2. Results of Mann–Whitney U tests comparing viral, microbial, and chemical variables between Arks and outplanting sites at each monitoring time point. Tests were conducted separately at each time point to evaluate treatment-level differences. Reported *P* values have been adjusted using the false discovery rate (FDR) method, with statistically significant values ($\alpha = 0.05$) shown in bold. Chemical variables are labeled using standard formulas for measured nutrients (e.g., PO_4^{3-}, NO_3^-, NH_4^+). Group means for each treatment and time point are also provided.

Variable	**Test type**	**Grouping**	**Time point**	**n**	**Mean Ark**	**Mean Outplant**	***P* value (FDR-adjusted)**
Virus-to-microbe ratio	MW	Time point	0	24	16.60	12.88	**< 0.001**
Virus-to-microbe ratio	MW	Time point	3	24	14.45	10.41	**< 0.001**
Virus-to-microbe ratio	MW	Time point	6	24	13.73	9.88	**< 0.001**
Virus-to-microbe ratio	MW	Time point	9	12	13.82	8.96	**0.011**
Virus-to-microbe ratio	MW	Time point	12	24	13.45	9.56	**< 0.001**
Virus-to-microbe ratio	MW	Time point	18	22	12.05	5.89	**0.001**
Virus-like-particles/mL	MW	Time point	0	24	8.61	6.67	**< 0.001**
Virus-like-particles/mL	MW	Time point	3	24	5.43	5.51	0.184
Virus-like-particles/mL	MW	Time point	6	24	6.29	5.74	**0.011**
Virus-like-particles/mL	MW	Time point	9	12	6.34	5.39	**0.016**
Virus-like-particles/mL	MW	Time point	12	24	8.83	5.84	**< 0.001**
Virus-like-particles/mL	MW	Time point	18	22	5.01	4.35	**0.011**
Microbial cells/mL	MW	Time point	0	24	5.20	5.18	0.737
Microbial cells/mL	MW	Time point	3	24	3.76	5.40	**0.001**
Microbial cells/mL	MW	Time point	6	24	4.60	5.83	**0.001**
Microbial cells/mL	MW	Time point	9	12	4.59	6.01	**0.011**
Microbial cells/mL	MW	Time point	12	24	6.63	6.22	0.495
Microbial cells/mL	MW	Time point	18	23	4.19	7.53	**0.001**
Microbial cell volume	MW	Time point	0	13	0.42	0.65	0.066
Microbial cell volume	MW	Time point	3	24	0.47	0.53	0.147
Microbial cell volume	MW	Time point	6	24	0.38	0.58	**0.001**
Microbial cell volume	MW	Time point	9	12	0.35	0.44	0.057
Microbial cell volume	MW	Time point	12	24	0.34	0.48	**0.001**
Microbial cell volume	MW	Time point	18	24	0.35	0.42	**0.011**
Total microbial biomass	MW	Time point	0	13	1.72	2.93	0.066
Total microbial biomass	MW	Time point	3	24	1.76	2.67	**0.004**
Total microbial biomass	MW	Time point	6	24	2.82	2.90	0.495
Total microbial biomass	MW	Time point	9	12	2.61	3.11	0.289
Total microbial biomass	MW	Time point	12	24	2.46	2.93	**0.043**
Total microbial biomass	MW	Time point	18	24	2.28	4.88	**0.001**

DOC	MW	Time point	3	24	96.58	80.82	**0.010**
DOC	MW	Time point	6	24	83.78	88.59	0.160
DOC	MW	Time point	9	11	85.02	102.70	**0.016**
DOC	MW	Time point	12	24	76.90	80.16	0.262
DOC	MW	Time point	18	24	100.46	109.66	0.559
PO_4^{3-}	MW	Time point	3	22	0.005	0.008	0.510
PO_4^{3-}	MW	Time point	6	24	0.007	0.001	0.289
PO_4^{3-}	MW	Time point	9	12	0.020	0.008	0.532
PO_4^{3-}	MW	Time point	12	24	0.048	0.048	0.878
PO_4^{3-}	MW	Time point	18	24	0.093	0.064	0.161
$NO_3^- + NO_2^-$	MW	Time point	3	22	0.074	0.057	0.289
$NO_3^- + NO_2^-$	MW	Time point	6	24	0.054	0.056	0.280
$NO_3^- + NO_2^-$	MW	Time point	9	12	0.230	0.333	0.354
$NO_3^- + NO_2^-$	MW	Time point	12	24	0.044	0.252	**0.001**
$NO_3^- + NO_2^-$	MW	Time point	18	24	0.206	1.688	**0.011**
NH_3	MW	Time point	3	22	0.078	0.202	**0.002**
NH_3	MW	Time point	6	24	0.020	0.041	0.183
NH_3	MW	Time point	9	12	0.193	0.165	0.935
NH_3	MW	Time point	12	24	0.186	0.222	0.117
NH_3	MW	Time point	18	24	0.203	0.131	0.100

Table S3. Results of Kruskal–Wallis tests evaluating temporal differences within treatments for each viral, microbial, and chemical variable. Tests were conducted separately for Arks and outplanting sites to assess differences across monitoring time points. Sample sizes (n) are reported for each test. *P* values were adjusted using the false discovery rate (FDR) method, with statistically significant values ($\alpha = 0.05$) shown in bold.

Variable	Test type	Grouping	Treatment	n	*P* value (FDR-adjusted)
Virus-to-microbe ratio	KW	Treatment	Ark	65	**< 0.001**
Virus-like-particles/mL	KW	Treatment	Ark	65	**< 0.001**
Microbial cells/mL	KW	Treatment	Ark	65	**< 0.001**
Microbial cell volume	KW	Treatment	Ark	65	**< 0.001**
Total microbial biomass	KW	Treatment	Ark	65	**< 0.001**
DOC	KW	Treatment	Ark	53	**< 0.001**
PO_4^{3-}	KW	Treatment	Ark	53	**< 0.001**
$NO_3^- + NO_2^-$	KW	Treatment	Ark	53	**0.008**
NH_3	KW	Treatment	Ark	53	**< 0.001**
Virus-to-microbe ratio	KW	Treatment	Outplant	65	**< 0.001**
Virus-like-particles/mL	KW	Treatment	Outplant	65	**< 0.001**
Microbial cells/mL	KW	Treatment	Outplant	66	**< 0.001**
Microbial cell volume	KW	Treatment	Outplant	56	**< 0.001**
Total microbial biomass	KW	Treatment	Outplant	56	**< 0.001**
DOC	KW	Treatment	Outplant	54	**< 0.001**
PO_4^{3-}	KW	Treatment	Outplant	53	**< 0.001**
$NO_3^- + NO_2^-$	KW	Treatment	Outplant	53	**< 0.001**
NH_3	KW	Treatment	Outplant	53	**< 0.001**

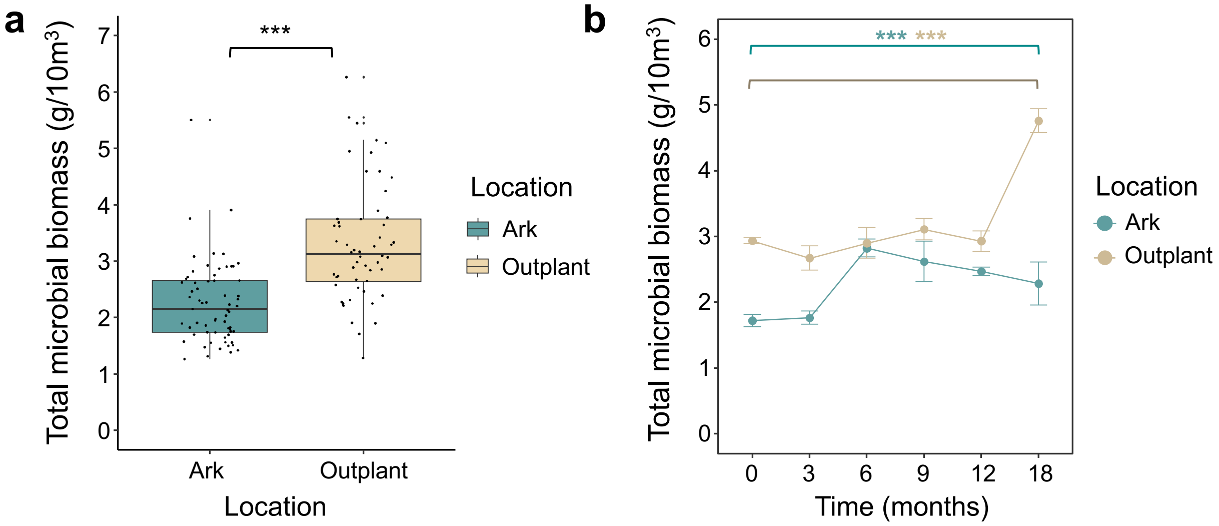

Figure S3. Boxplots showing total microbial biomass of seawater microbial communities collected from the Arks and outplanting sites for all timepoints (left). Asterisks denote significant differences between Ark and outplant treatments (Mann Whitney U test, $P < 0.001$). Line plot showing the same variable plotted over time (right). Colored brackets and asterisks denote significant differences within the Ark (teal) and outplanting (tan) treatments over time (Kruskal-Wallis test, $P < 0.001$).

Table S4. Mann–Whitney U test results comparing physical parameters between Arks and outplanting sites at each monitoring time point. Variables include water flow, light intensity (daytime only), temperature, and dissolved oxygen. Group means, sample sizes (*n*), and FDR-adjusted *P* values are reported for each time point. Statistically significant results (α = 0.05) are shown in bold.

Variable	**Test type**	**Grouping**	**Time point**	**MW Statistic**	**Mean Ark**	**Mean Outplant**	**n Ark**	**n Outplant**	***P* value (FDR-adjusted)**
Flow	MW	Time point	0	193418.5	11.57	6.35	814	289	**< 0.001**
Flow	MW	Time point	6	21750698	13.40	8.09	10057	2631	**< 0.001**
Flow	MW	Time point	9	7958004.5	9.57	6.32	2759	3989	**< 0.001**
Flow	MW	Time point	18	6364591	15.91	8.90	3019	2369	**< 0.001**
Light intensity	MW	Time point	0	1661125	7807.84	2318.92	1584	1290	**< 0.001**
Light intensity	MW	Time point	3	910873.5	11951.70	7249.16	1188	1188	**< 0.001**
Light intensity	MW	Time point	6	1628260.5	13919.56	5707.61	1452	1452	**< 0.001**
Light intensity	MW	Time point	9	1289980.5	11581.64	8050.49	1452	1452	**< 0.001**
Light intensity	MW	Time point	12	1369947	6534.50	4108.41	1452	1452	**< 0.001**
Temperature	MW	Time point	0	5407445	28.79	28.82	3456	2808	**< 0.001**
Temperature	MW	Time point	3	2792769	27.02	27.07	2655	2655	**< 0.001**
Temperature	MW	Time point	6	3688882.5	28.04	28.18	3169	3168	**< 0.001**
Temperature	MW	Time point	9	4658542.5	29.85	29.87	3168	3168	**< 0.001**
Temperature	MW	Time point	12	5860683.5	27.53	27.48	3170	3170	**< 0.001**
Dissolved oxygen	MW	Time point	0	2929435.5	6.27	6.19	4512	4512	**< 0.001**
Dissolved oxygen	MW	Time point	3	21549904	6.45	6.14	11866	11866	**< 0.001**

Dissolved oxygen	MW	Time point	6	125596929	6.47	6.41	28926	28926	**< 0.001**
Dissolved oxygen	MW	Time point	9	117420308.5	6.05	5.99	28196	28196	**< 0.001**
Dissolved oxygen	MW	Time point	12	100627989.5	6.29	6.11	23991	23991	**< 0.001**
Dissolved oxygen	MW	Time point	18	42024391	6.12	6.01	17058	17058	**< 0.001**

Table S5. Results of Kruskal–Wallis tests comparing physical parameters between Arks and outplanting sites across all monitoring time points. Variables include water flow, light intensity (daytime only), temperature, and dissolved oxygen. Degrees of freedom (df), group means, and FDR-adjusted *P* values are reported. Statistically significant differences (α = 0.05) are shown in bold.

Variable	**Test type**	**Grouping**	**KW Chi Square**	**df**	**Mean Ark**	**Mean Outplant**	***P* value (FDR-adjusted)**
Flow	KW	All time	7885.82	1	12.98	7.47	**< 0.001**
Light Intensity	KW	All time	1396.01	1	10252.81	5493.94	**< 0.001**
Temperature	KW	All time	0.09	1	28.29	28.31	0.762
Dissolved Oxygen	KW	All time	4198.92	1	6.26	6.15	**< 0.001**

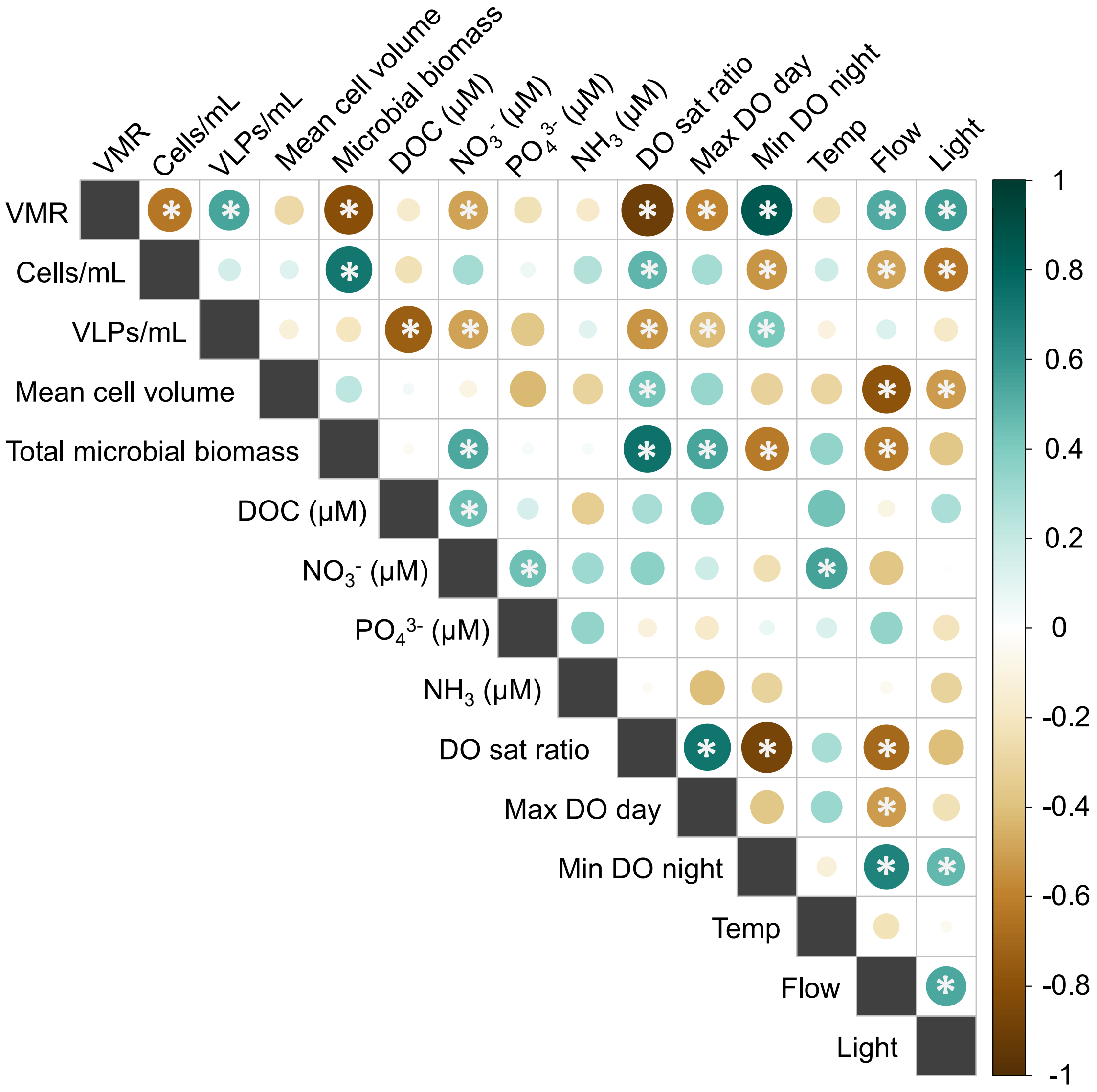

Figure S4. Relationships between viral and microbial, chemical, and physical variables. Spearman's correlation matrix, with green colors indicating a positive correlation and brown colors indicating a negative correlation between each variable pair (darker color = stronger correlation). Circle size indicates significance of the correlation, with an asterisk denoting values below a significance threshold of $P < 0.05$. DO Sat Ratio refers to the ratio of daytime DO measurements to nighttime DO measurements, with larger values indicating higher diel variance in dissolved oxygen.

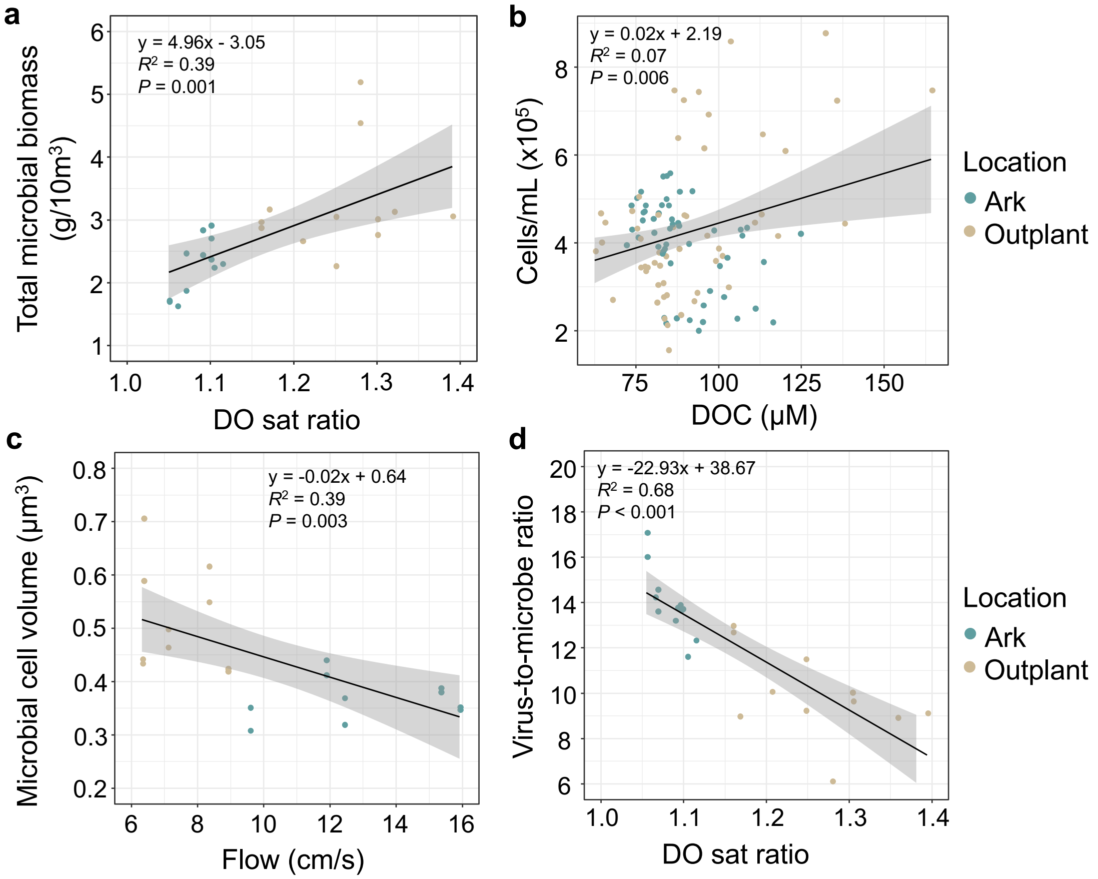

Figure S5. Linear regression models of select viral, microbial, chemical, and physical variables at Arks and outplanting sites with linear equations, R^2, and *P* value (t-test) included. (a) Total microbial biomass vs. DO Sat Ratio. (b) Microbial cell abundance vs dissolved organic carbon (DOC) concentration. (c) Mean microbial cell size vs flow speed. (d) Virus-to-microbe ratio vs DO Sat Ratio. DO Sat Ratio refers to the ratio of daytime DO measurements to nighttime DO measurements, with larger values indicating higher diel variance.

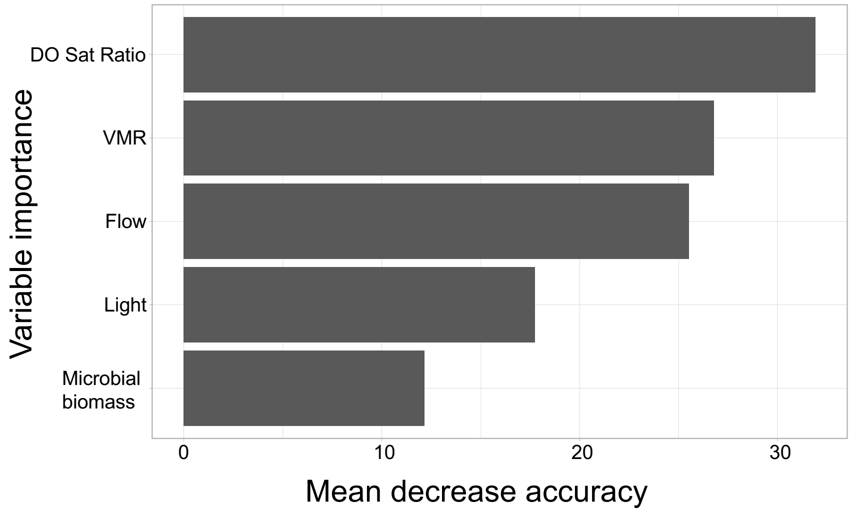

Figure S6. Results from a permuted random forest to determine significant predictive variables of whether measurements were collected from the Arks or outplant sites.

Table S6. Results from Type III ANOVA performed on linear models evaluating central carbon metabolism gene pathway abundances in microbial communities at Arks and outplanting sites over time. Values represent FDR-adjusted *P* values for each model term, with bolded values indicating significant differences at a threshold of α = 0.05. EMP = Embden-Meyerhof-Parnas; ED = Entner-Doudoroff; PP = Pentose Phosphate Pathway. **Within-treatment time trends** assess change in gene abundance over time within each treatment group, with shading denoting the directionality of the trend over time: tan for increasing, teal for decreasing. **Treatment effect tests** compare mean gene abundance between Ark and outplanting treatments, averaged across time. **Interaction tests** evaluate whether temporal trends differ between treatments (i.e., whether slopes over time vary between Ark and outplanting sites).

Variable tested/ Gene pathway	Anaplerotic Rxns	EMP Pathway	ED Pathway	PP Pathway	Oxidative Krebs cycle	Rxns fueling ED and PPP
Ark (across time)	***P* < 0.001**	***P* < 0.001**	***P* < 0.001**	***P* < 0.001**	***P* < 0.001**	***P* < 0.001**
Outplant (across time)	***P* < 0.001**	***P* < 0.001**	***P* < 0.001**	***P* < 0.001**	***P* < 0.001**	***P* < 0.001**
Ark vs outplant (treatment effect over all time)	***P* < 0.001**	***P* < 0.001**	***P* < 0.001**	***P* < 0.001**	***P* < 0.001**	***P* < 0.001**
*Interaction effect (time * treatment)*	0.330	0.544	0.594	**0.007**	0.544	**0.018**

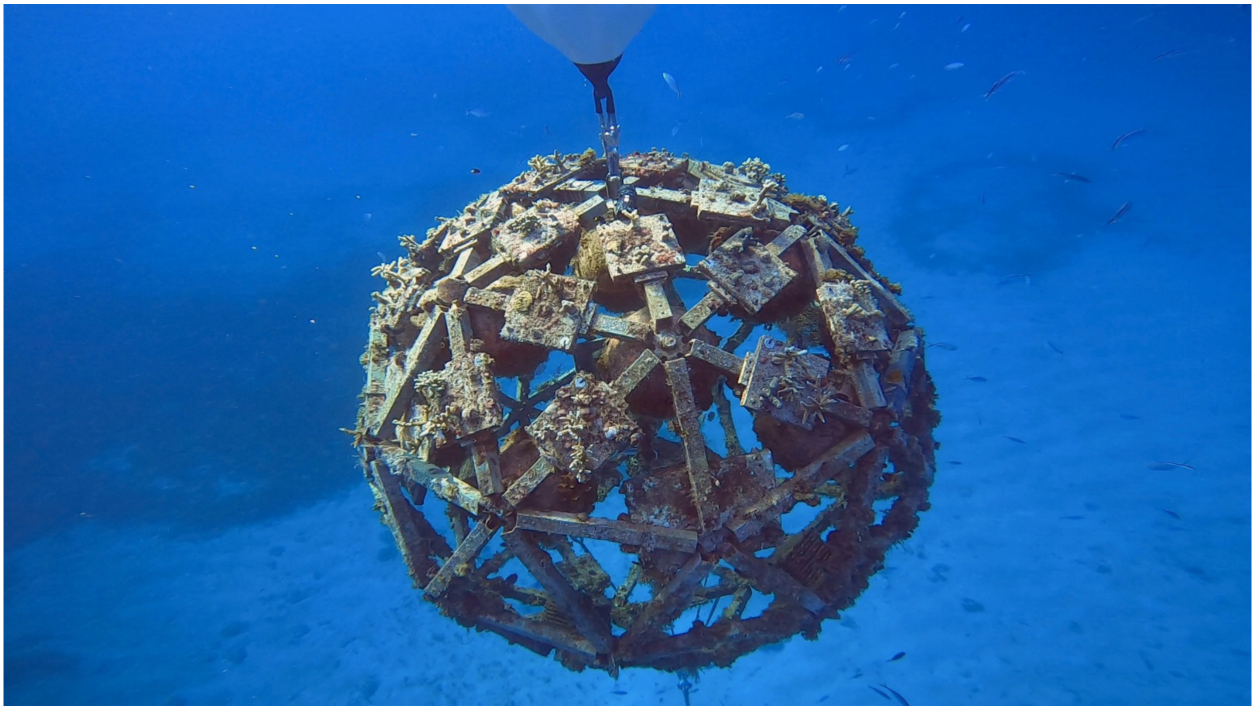

Figure S7. Photograph of a Coral Ark at the 12-month monitoring time point. The image shows an Ark structure suspended in the water column, with visible coral growth and associated reef organisms illustrating the development of a diverse benthic community over the course of the experiment.

Table S7. Top 20 most abundant bacterial genera detected in metagenomic sequencing data from Ark and outplanting sites. Total relative abundance values (%) are reported for each genus at both site types, ranked by overall abundance across all samples.

Treatment	Kingdom	Phylum	Class	Order	Family	Genus	Total Relative Abundance (%)
Ark	Bacteria	Proteobacteria	Alphaproteobacteria	Unknown Order	Unknown Family	alpha_proteobacterium	13.6
Ark	Bacteria	Cyanobacteria	Unknown Class	Prochlorales	Prochlorococcaceae	Prochlorococcus	4.9
Ark	Bacteria	Tenericutes	Mollicutes	Mycoplasmatales	Mycoplasmataceae	Mycoplasma	3.1
Ark	Bacteria	Cyanobacteria	Unknown Class	Chroococcales	Unknown Family	Synechococcus	3.0
Ark	Archaea	Euryarchaeota	Halobacteria	Halobacteriales	Halobacteriaceae	Haloquadratum	1.9
Ark	Bacteria	Bacteroidetes	Flavobacteriia	Flavobacteriales	Flavobacteriaceae	Flavobacterium	1.9
Ark	Bacteria	Bacteroidetes	Cytophagia	Cytophagales	Cyclobacteriaceae	Belliella	1.2
Ark	Bacteria	Thermotogae	Thermotogae	Thermotogales	Thermotogaceae	Thermotoga	1.0
Ark	Bacteria	Proteobacteria	Alphaproteobacteria	Rhodobacterales	Rhodobacteraceae	Octadecabacter	0.9
Ark	Bacteria	Proteobacteria	Gammaproteobacteria	Oceanospirillales	Oceanospirillaceae	Marinomonas	0.9
Ark	Bacteria	Tenericutes	Mollicutes	Entomoplasmatales	Spiroplasmataceae	Spiroplasma	0.8
Ark	Bacteria	Spirochaetes	Spirochaetia	Spirochaetales	Leptospiraceae	Leptospira	0.8
Ark	Archaea	Thaumarchaeota	Unknown Class	Nitrosopumilales	Nitrosopumilaceae	Nitrosoarchaeum	0.7
Ark	Bacteria	Proteobacteria	Gammaproteobacteria	Unknown Order	Unknown Family	Carsonella	0.7
Ark	Bacteria	Proteobacteria	Alphaproteobacteria	Rickettsiales	Anaplasmataceae	Anaplasma	0.7
Ark	Bacteria	Proteobacteria	Alphaproteobacteria	Rhodobacterales	Hyphomonadaceae	Hirschia	0.7
Ark	Bacteria	Actinobacteria	Actinobacteria	Actinomycetales	Jonesiaceae	Jonesia	0.7
Ark	Bacteria	Proteobacteria	Epsilonproteobacteria	Campylobacterales	Helicobacteraceae	Helicobacter	0.6
Ark	Bacteria	Proteobacteria	Alphaproteobacteria	Rickettsiales	Anaplasmataceae	Ehrlichia	0.6
Control	Bacteria	Proteobacteria	Alphaproteobacteria	Unknown Order	Unknown Family	alpha_proteobacterium	10.7
Control	Bacteria	Cyanobacteria	Unknown Class	Chroococcales	Unknown Family	Synechococcus	4.0
Control	Bacteria	Cyanobacteria	Unknown Class	Prochlorales	Prochlorococcaceae	Prochlorococcus	2.8
Control	Bacteria	Proteobacteria	Alphaproteobacteria	Rhodobacterales	Rhodobacteraceae	Octadecabacter	2.7
Control	Bacteria	Proteobacteria	Alphaproteobacteria	Rhodobacterales	Hyphomonadaceae	Hirschia	2.2
Control	Bacteria	Tenericutes	Mollicutes	Mycoplasmatales	Mycoplasmataceae	Mycoplasma	1.9
Control	Bacteria	Bacteroidetes	Cytophagia	Cytophagales	Cyclobacteriaceae	Belliella	1.5
Control	Bacteria	Bacteroidetes	Flavobacteriia	Flavobacteriales	Flavobacteriaceae	Flavobacterium	1.5
Control	Archaea	Euryarchaeota	Halobacteria	Halobacteriales	Halobacteriaceae	Haloquadratum	1.3
Control	Bacteria	Actinobacteria	Actinobacteria	Actinomycetales	Jonesiaceae	Jonesia	1.0
Control	Bacteria	Firmicutes	Bacilli	Bacillales	Staphylococcaceae	Staphylococcus	0.8
Control	Bacteria	Proteobacteria	Gammaproteobacteria	Oceanospirillales	Oceanospirillaceae	Marinomonas	0.8
Control	Archaea	Thaumarchaeota	Unknown Class	Nitrosopumilales	Nitrosopumilaceae	Nitrosoarchaeum	0.8
Control	Bacteria	Spirochaetes	Spirochaetia	Spirochaetales	Leptospiraceae	Leptospira	0.7
Control	Bacteria	Thermotogae	Thermotogae	Thermotogales	Thermotogaceae	Thermotoga	0.6
Control	Bacteria	Proteobacteria	Alphaproteobacteria	Rhodobacterales	Rhodobacteraceae	Ruegeria	0.6
Control	Bacteria	Actinobacteria	Actinobacteria	Actinomycetales	Unknown Family	Tropheryma	0.6
Control	Bacteria	Bacteroidetes	Flavobacteriia	Flavobacteriales	Flavobacteriaceae	Muricauda	0.6
Control	Bacteria	Proteobacteria	Gammaproteobacteria	Unknown Order	Unknown Family	Carsonella	0.6
